# An Interesting Case of Neonatal AKI: What Is the Time to Consider Anuria Irreversible?

**DOI:** 10.3390/children10061032

**Published:** 2023-06-08

**Authors:** Antonio Gatto, Eloisa Tiberi, Serena Ferretti, Valerio Santoro, Alessandra Piersanti, Filomena Valentina Paradiso, Lorenzo Nanni, Roberto Iezzi, Alessandro Posa, Simonetta Costa, Giovanni Vento

**Affiliations:** 1 Department of Pediatrics, Fondazione Policlinico Universitario “A. Gemelli” IRCCS, 00168 Rome, Italy; serena.ferretti01@icatt.it; 2 Neonatology Unit, Department of Woman and Child Health and Public Health, Fondazione Policlinico Universitario “A. Gemelli” IRCCS, 00168 Rome, Italy; eloisa.tiberi@policlinicogemelli.it (E.T.); giovanni.vento@unicatt.it (G.V.); 3 Department of Pediatrics, Università Cattolica del Sacro Cuore, 00168 Rome, Italy; valerio.santoro01@icatt.it; 4 Neonatology Unit, Università Cattolica del Sacro Cuore, 00168 Rome, Italy; piersanti.ap@gmail.com (A.P.); simonetta.costa@policlinicogemelli.it (S.C.); 5 Pediatric Surgery Unit, Department of Woman and Child Health and Public Health, Fondazione Policlinico Universitario “A. Gemelli” IRCCS, 00168 Rome, Italy; filomenavalentina.paradiso@policlinicogemelli.it (F.V.P.); lorenzo.nanni@policlinicogemelli.it (L.N.); 6 Department of Radiology, Fondazione Policlinico Universitario “A. Gemelli” IRCCS, 00168 Rome, Italy; roberto.iezzi@policlinicogemelli.it (R.I.); alessandro.posa@policlinicogemelli.it (A.P.)

**Keywords:** acute kidney injury, extremely low birth weight, peritoneal dialysis, prematurity

## Abstract

Acute kidney injury is a frequent complication for critical newborns. Its management is a significant challenge, especially in extremely low-birth-weight (ELBW) infants. Currently, peritoneal dialysis (PD) is the most manageable treatment. However, data are lacking regarding when diuresis can be declared irreversible relative to the start of PD. A female infant born at 28 + 0 weeks with a birth weight of 800 g by monochorionic diamniotic pregnancy, complicated by twin-to-twin transfusion syndrome, developed acute renal failure on the second day of life because of long-term intrauterine hypoperfusion. PD was started on day 7. The patient remained anuric until the 52nd day of dialysis, when she presented adequate urine output of 2.5 mL/kg/h and PD was suspended for 11 days. After an episode of sepsis, PD was re-started, and after 50 days of treatment, given a urine output of 1.5 mL/kg/h, it was discontinued. The patient died on day 132 after a disseminate infection, which led to multiorgan failure. In ELBW infants, PD is a valid therapeutic instrument to treat patients with renal failure. Despite the evidence of low renal functional reserve in these patients, the duration of recovery from diuresis after a period of anuria can be very long.

## 1. Introduction

Acute kidney injury (AKI) is a frequent complication in critically ill newborns, with an incidence of 8–24% [1]. Prematurity, perinatal asphyxia, sepsis, respiratory distress syndrome, and patent ductus arteriosus may cause it. AKI management is still a challenge, especially in case of extremely premature newborns, and it has an important impact on the survival rates of these neonates: the mortality of extremely low-birth-weight (ELBW) infants, in the case of AKI, reaches 78% [1,2,3]. When pharmacological management proves insufficient, the most manageable treatment for these infants is peritoneal dialysis (PD), which is associated with careful control of electrolytes, fluids, acid–base balance, nutrition, and the avoidance of infectious complications.

When considering RRT, the low weights of these children present strong challenges, such as limited availability of dialysis catheters, difficult catheter placement, and lack of access to machines available for newborns [4]. Therefore, the PD approach is used to successfully provide dialysis to these infants, although it is not a risk-free technique: complications of PD include catheter obstruction, bowel perforation with catheter insertion, catheter exit site leakage or infection, hypoalbuminemia, hyperglycemia, and peritonitis [4].

There are no data to support the time frame in which diuresis can reasonably be declared irreversible from the onset of PD in premature infants. We report our experience with PD in an ELBW infant whose diuresis restarted for the first time after 52 days of dialytic treatment.

## 2. Case Report

A female infant was born at 28 + 0 weeks of gestational age (GA) with a birth weight of 800 g after an emergency cesarean section. The pregnancy was monochorionic and diamniotic, complicated by twin-to-twin transfusion syndrome (TTTS). The patient was the donor twin; the recipient twin died of heart failure soon after birth. She had a prenatal condition of anhydramnios, an empty bladder with normal doppler assessment (Stage II TTTS). The APGAR score was 3 at 1 min and 6 at 5 min. In the delivery room, the newborn required intubation and mechanical ventilation was started according to the American Academy of Pediatrics’ guidelines. After 6 h in the Neonatal Intensive Care Unit (NICU), considering the diagnosis of respiratory distress syndrome, the infant was treated with 200 mg/kg of poractant alfa (Curosurf). After surfactant administration, the baby was extubated within 30 min, and non-invasive ventilation was started with nasal continuous positive airway pressure (nCPAP).

The kidney function at birth was good, with a diuresis of 1.6 mL/kg/h during the first 24 h of life and 1.3 mg/dL serum creatinine (Scr). On the 2nd day of life (DOL), she became anuric, with worsening kidney function tests (Scr 2.67 mg/dL and blood urea nitrogen 57 mg/dL). A renal Doppler ultrasound evaluation, performed on the 4th DOL, showed reduced kidney size for GA and increased intraparenchymal resistance. In accordance with the status of the donor in the TTTS, she developed acute renal failure that may have been caused by long-term intrauterine hypoperfusion resulting in severe delayed kidney development [5].

Anuria was unresponsive to pharmacological treatment (albumin 1 g/kg/die, furosemide 2 mg/kg every 8 h and fenoldopam 0.5 mcg/kg/min). Scr and blood urea nitrogen (BUN) were found to be 4.69 mg/dL and 98 mg/dL on 7th DOL, respectively. The newborn developed progressive edema and a 33% fluid weight gain from birth. Given her worsening clinical condition, the electrolyte disequilibrium, and the generalized edema, PD was started on DOL 7 [6].

A neonatal straight 10 Fr single-cuff Foley catheter (rush amber silicone-treated) was inserted with a left paramedian entry site in the left iliac fossa and advanced along the peritoneum with the distal end placed in the pelvis by a pediatric surgeon. A Tenckhoff catheter could not be used because of the low weight of the child. Treatment with PD fluids (2.3%) was started at a rate of 10 mL/kg, which was increased to 20–30 mL/kg at 60–120 min/cycle and continued for 24 h based on the effect of the dialysis.

The infant’s condition improved, with decreased Scr and BUN levels (2.6 mg/dL and 23 mg/dL, respectively), on day 10 of PD.

The patient remained anuric until the 52nd day of PD, when she presented an adequate urine output of 2.5 mL/kg/h and PD was suspended for 11 days. Due to the worsening of the clinical conditions caused by an episode of sepsis (*S. epidermidis*, documented by positive blood culture and treated with meropenem and vancomycin with dosage adjusted for renal function and dialysis treatment), PD was re-started with a mono-j straight 6F catheter positioned at the same site as previously with an emergency procedure. This was due to multi-organ failure, which made it impossible to perform the standard surgical procedure of inserting a Tenckhoff catheter. After 50 days of treatment, given the good clinical conditions, adequate urine output of 1.5 mL/kg/h, and good electrolyte balance and pH, PD was discontinued.

**Figure 1:**
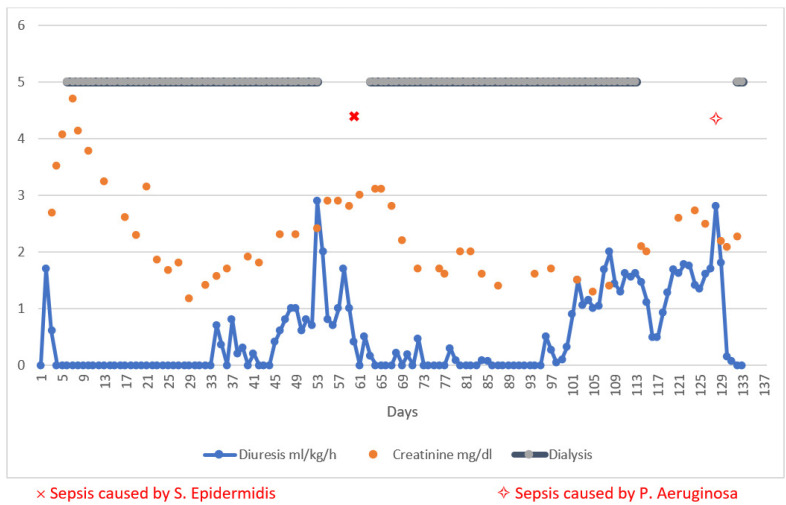
Trend of creatinine and diuresis during peritoneal dialysis.

The patient’s lowest serum creatinine level was 1.3 mg/dL, detected on the 104th DOL, when the infant weighed 2050 g. Due to the clinical worsening caused by a peritoneal and urinary tract infection (*P. aeruginosa*, documented by positive peritoneal fluid and urine culture), treated with meropenem and ciprofloxacin with dosage adjusted for renal function and dialysis treatment, on the 131st DOL, PD was restarted. The Scr and diuresis trends are shown in Figure 1.

In addition to peritonitis, leakage was another complication during the dialysis. However, it did not compromise the efficacy of PD.

Furosemide 1 mg/kg; fenoldopam 0.5 mcg/kg/min; dopamine 3–5 mcg/kg/min; ethacrynic acid 0.1 mg/kg/h; albumin 1 g/kg/die infusion; and bolus injections of HCO_3_^−^ and electrolytes modulated according to serum K, Na, and HCO_3_^−^ levels were administered for renal dysfunction. The dosages of the antibiotics were re-arranged according to the estimated creatinine clearance.

Renal ultrasonography was performed several times during the patient’s hospital stay, and all revealed a reduced kidney size for GA with increased echogenicity, loss of corticomedullary differentiation, and increased intraparenchymal resistance. The patient was never discharged; she requires mechanical ventilation for 86 days and non-invasive ventilation for 46 days of life, while nutrition was maintained parenterally due to feeding intolerance. The patient died on DOL 132 after a disseminate infection (*P. aeruginosa*, documented by positive blood and peritoneal fluid culture), which led to multiorgan failure that was unresponsive to inotropic and maximal supportive therapy.

## 3. Discussion

The kidney is one of the most vulnerable organs in preterm infants. The incomplete nephrogenesis process and the limited number of nephrons expose ELBW newborns to an increased susceptibility to AKI, also considering the higher risk of infections and the exposure to nephrotoxic medications typical of NICU care [7]. The early identification of risk factors and the close monitoring of kidney function markers, diuresis, electrolytes, and acid–base balance are crucial for a timely and effective pharmacological intervention and, if necessary, replacement therapy. Data on PD in ELBW infants in the literature are limited, but PD is currently considered as the only rescue therapy for ELBW newborns affected by AKI, because there are no data regarding the efficacy of hemodialysis (HD) in this population [8]. Moreover, hemodialysis is extremely difficult perform in ELBW, because there is no small vascular catheter for HD for these children, and data regarding HD machines that can efficiently handle such low-exchange blood volumes are still extremely meager [4,9].

The high rates of complications (25–60%) and mortality (>50%) described by the authors are the main criticalities of the procedure for these patients [10]. The current unavailability of an adequately sized peritoneal catheter for ELBW infants represents an additional challenge. Furthermore, the thin abdominal wall, which does not allow for adequate subcutaneous tunneling of the catheter in these patients, can lead to a real risk of failure of the procedure due to a feasibility problem, which also poses a risk of infection. Another difficult management factor is the absence of data regarding the optimal PD regimen for very-low-birth-weight and ELBW infants, with the consequent need for strict personalized management of the case by the nephrologist in synergy with the neonatologist [11].

The case which we report herein seems to be a typical AKI in ELBW newborn, with TTTS as the known risk factor causing prenatal kidney hypoperfusion. It highlights, as do the other cases available in the literature, the absolute necessity of preserving the kidney and its functional reserve using all the available means. Moreover, the clinical course, according to the infectious, ventilatory, and metabolic profiles of the infant, is superimposable to similar reported cases (Supplementary Material—Table S1).

In fact, we performed an analysis of the currently available literature of case reports and case series of newborns born at GA ≤ 28 weeks with a birth weight ≤ 1000 g who underwent PD. All relevant data are recorded in Table S1. The median GA was 26 weeks, and the median birth weight was 700 g. Among the 48 cases described, 18 (37.5%) recovered normal kidney function, 29 (60.4%) died, and 1 (2.1%) survived with chronic kidney disease at stage 4. PD was performed in these children for a mean time of 2 days and a median time of 4.7 days.

However, the surprising aspect of the clinical case described in this article is the timing that the diuresis was restarted. The girl had a first period of 52 days of oligo-anuria and a second one of 50 days, with several infectious episodes, coagulopathy phases, and device complications. However, in periods of well-being and when PD was stopped, diuresis was normal (>1 mL/kg/die), although improvements of kidney vascularization on serial Doppler ultrasound assessments were absent. Considering these data, it is useful to wonder whether there is actually a time limit to declare the failure of the dialysis treatment, and whether the functional renal reserve of an ELBW newborn can guarantee the possibility for diuresis to be restarted even after a very long period of anuria. Currently, there is no shared opinion on this point available in the literature, and no author has attempted to use a time reference as a guideline.

Furthermore, from a decision-making and, consequently, an ethical point of view, the ability of the kidney to resume its functionality after a long time also entails a reflection on the need to continue dialysis, without losing sight of the concept of proportionality of the treatments.

We believe that describe reporting of these cases is useful to increase the data on this population, which, with the improvement of assistance and survival, will be more numerous in the coming years. Indications and methods of PD for ELBW infants with AKI should be described in guidelines in order to reduce complications and to optimize and standardize the timing at which the treatment begins.

We highlight the technical challenge represented in our case by the use of the 6F straight mono-j catheter. This represents a very interesting novelty for patients in the NICU, who are often in such critical condition that they cannot undergo a standard surgical procedure, or are too small to receive a larger catheter.

## 4. Conclusions

Our case demonstrated, once again, that in ELBW, PD is a valid therapeutic instrument to treat patients with renal failure. Despite the evidence of low renal functional reserve in these patients, the duration of the recovery from diuresis after a period of anuria can be very long. From an ethical point of view, given the extreme fragility of ELBW newborns and the high rate of complications related to prematurity, the proportionality of PD should be constantly evaluated during dialytic treatment.

## Data Availability

All the relevant data are described in the main text.

## Supplementary Materials

The following supporting information can be downloaded at: https://www.mdpi.com/article/10.3390/children10061032/s1, Table S1: Case reports of children with a birth weight ≤ 1000 g and a gestational age ≤ 28 weeks who underwent peritoneal dialysis currently available in the literature.
