# Supplementary material for: An Interesting Case of Neonatal AKI: What Is the Time to Consider Anuria Irreversible?
Source: Children (Basel). 2023 Jun 8;10(6):1032. doi: 10.3390/children10061032 (PMC10296958; doi:10.3390/children10061032)
Supplement: Supplementary file 1 [file children-10-01032-s001.zip › children-2412662-supplementary.pdf]

Authors	Cause of AKI	GA (weeks)	BW (g)	Catheter type/ insertion site	Age at the beginning of PD (days)/PD solution	Duration of PD (days)	Complications	Outcome
**Kanarek et al. (1981)**	Asphyxia, acute tubular necrosis	25	710	Trocath-McGaw catalog #V4900/ND	2/ND	30 hours	None	Recovered
**Sizun et al. (1993)**	Sepsis	28	680	Venflon Viggo 16-gauge peripheral venous catheter/ND	41/1.36% isotonic Dianeal glucose solution	2	None	Died (nonrenal)
**Sizun et al. (1993)**	Asphyxia	25	700	Venflon Viggo 16-gauge peripheral venous catheter/ND	5/1.36% isotonic Dianeal glucose solution	3	None	Died (nonrenal)
**Yu et al. (2009)**	Sepsis	26	930	14-gauge Arrow vascular catheter/Tip in controlateral iliac fossa	10/Dianeal glucose solution at different concentrations+1 UI/mL heparin	2	Hernia	Recovered
**Yu et al. (2009)**	PDA	26	890	14-gauge Arrow vascular catheter/Tip in controlateral iliac fossa	25/Dianeal glucose solution at different concentrations+1 UI/mL heparin	2	Peritonitis	Recovered
**Yu et al. (2009)**	Sepsis	28	900	14-gauge Arrow vascular catheter/Tip in controlateral iliac fossa	14/Dianeal glucose solution at different concentrations+1 UI/mL heparin	6	None	Recovered
**Yu et al. (2009)**	Sepsis	28	730	14-gauge Arrow vascular catheter/Tip in controlateral iliac fossa	26/Dianeal glucose solution at different concentrations+1 UI/mL heparin	3	None	Recovered

**Yu et al. (2009)**	PDA	26	680	14-gauge Arrow vascular catheter/Tip in controlateral iliac fossa	28/Dianeal glucose solution at different concentrations+1 UI/mL heparin	6	None	Recovered
**Yu et al. (2009)**	Pulmonary hemorrhage	27	690	14-gauge Arrow vascular catheter/Tip in controlateral iliac fossa	8/Dianeal glucose solution at different concentrations+1 UI/mL heparin	8	None	Recovered
**Yu et al. (2009)**	Intracranial hemorrage	26	700	14-gauge Arrow vascular catheter/Tip in controlateral iliac fossa	21/Dianeal glucose solution at different concentrations+1 UI/mL heparin	2	Leakage	Died
**Yu et al. (2009)**	Sepsis	28	980	14-gauge Arrow vascular catheter/Tip in controlateral iliac fossa	32/Dianeal glucose solution at different concentrations+1 UI/mL heparin	3	Peritonitis	Died
**Yu et al. (2009)**	Necrotizing enterocolitis	26	840	14-gauge Arrow vascular catheter/Tip in controlateral iliac fossa	18/Dianeal glucose solution at different concentrations+1 UI/mL heparin	2	Leakage, hemoperitoneum	Died
**Yu et al. (2009)**	Pulmonary hemorrhage	26	630	14-gauge Arrow vascular catheter/Tip in controlateral iliac fossa	27/Dianeal glucose solution at different concentrations+1 UI/mL heparin	2	None	Died (nonrenal)
**Yu et al. (2009)**	PDA	24	820	14-gauge Arrow vascular catheter/Tip in controlateral iliac fossa	26/Dianeal glucose solution at different concentrations+1 UI/mL heparin	3	None	Died (nonrenal)

**Alparslan et al. (2012)**	RDS	28	1000	Tenckhoff, one-cuffed neonatal catheters with straight tips/Through the linea alba toward the left iliac fossa	ND/Dianeal PD-2 1.36-3.86%+heparin 500U/L+potassium chloride	12	None	Died
**Macchini et al. (2013)**	Sepsis	28	630	single-cuff Tenckhoff catheter/ paramedian entry-site	10/ND	27	Leakage	Recovered
**Harshman et al. (2014)**	TTTS	28+3/7	830	PD catheter/left upper quadrant	5/1.5% later 2.5% Dianeal	16	None	Recovered
**Stojanović et al. (2016)**	Sepsis	25	690	IV cannula/left side of umbilicus	6/4.25% or 2.5% and 1.5% afterwards (Dianeal)	3 hours	None	Died
**Stojanović et al. (2016)**	Sepsis	27+1/7	470	IV cannula/left side of umbilicus	4/4.25% or 2.5% and 1.5% afterwards (Dianeal)	2.5 hours	Leakage	Died
**Stojanović et al. (2016)**	Gentamicin	27+3/7	890	IV cannula/left side of umbilicus	17/4.25% or 2.5% and 1.5% afterwards (Dianeal)	28 hours	None	Died
**Stojanović et al. (2016)**	Sepsis	27	880	Umbilical venous catheter/ left side of umbilicus	28/4.25% or 2.5% and 1.5% afterwards (Dianeal)	2	Peritonitis	Died
**Stojanović et al. (2016)**	Sepsis	27	610	IV cannula/left side of umbilicus	11/4.25% or 2.5% and 1.5% afterwards (Dianeal)	4 hours	Obstruction	Died
**Stojanović et al. (2016)**	Necrotizing enterocolitis, PDA	25	880	IV cannula/left side of umbilicus	2/4.25% or 2.5% and 1.5% afterwards (Dianeal)	21 hours	None	Recovered

**Stojanović et al. (2016)**	Apnea	25	870	IV cannula/left side of umbilicus	13/4.25% or 2.5% and 1.5% afterwards (Dianeal)	1.5 hours	Leakage	Died
**Stojanović et al. (2016)**	Asphyxia	25	700	IV cannula/left side of umbilicus	20/4.25% or 2.5% and 1.5% afterwards (Dianeal)	22 hours	None	Recovered
**Ustyol et al. (2016)**	Sepsis	26	600	PD catheter/0.5-1cm below the umbilicus	14/1.36% or 2.27% Baxter	3	ND	Died
**Ustyol et al. (2016)**	Sepsis	25	750	PD catheter/0.5-1cm below the umbilicus	11/1.36% or 2.27% Baxter	1	ND	Died
**Ustyol et al. (2016)**	Sepsis	24	580	PD catheter/0.5-1cm below the umbilicus	7/1.36% or 2.27% Baxter	3	ND	Died
**Ustyol et al. (2016)**	Sepsis	24	700	PD catheter/0.5-1cm below the umbilicus	8/1.36% or 2.27% Baxter	1	ND	Died
**Yokoyama et al. (2017)**	Sepsis	24	264	Drainage catheter/right umbilical region	21/1.5% dialysate	32	Leakage, Peritonitis	Recovered
**Ao et al. (2018)**	Sepsis	24+3/7	700	14-gauge central venous catheter/1cm lateral left of the umbilicus	1/Dianeal PD-4	14 hours	ND	Recovered
**Ao et al. (2018)**	ARDS	24+6/7	750	14-gauge central venous catheter/1cm lateral left of the umbilicus	3/Dianeal PD-4	1.5	ND	Recovered
**Ao et al. (2018)**	Asphyxia	27+1/7	880	14-gauge central venous catheter/1cm	1/Dianeal PD-4	2	ND	Recovered

				lateral left of the umbilicus				
**Kaul et al. (2019)**	Necrotizing enterocolitis	27	960	Intercostal drain (modified)/ left paraumbilical region	40/1.7% dialysate	2	None	Recovered
**Çetinkaya et al. (2020)**	Sepsis	24	460	PD catheter/left paramedian above umbilicus	12/1.36% dialysate	6	Leakage	Died
**Noh et al. (2020)**	Sepsis	25+1/7	960	PD catheter/McBurney point	8/Hemosol or Physioneal (standard hydrous dextrose) from 2.5% to 4.25%	9.4 (5-14)	Leakage, intraperitoneal hemorrhage, obstruction	Died
**Noh et al. (2020)**	MODS	26+4/7	990	UV catheter/McBurney point	3/Hemosol or Physioneal (standard hydrous dextrose) from 2.5% to 4.25%	9.4 (5-14)	Peritonitis, intraperitoneal hemorrhage	Died
**Noh et al. (2020)**	Sepsis	25+2/7	420	ARROW catheter/McBurney point	43/Hemosol or Physioneal (standard hydrous dextrose) from 2.5% to 4.25%	9.4 (5-14)	None	Died
**Noh et al. (2020)**	Sepsis	24+2/7	540	ARROW catheter/McBurney point	26/Hemosol or Physioneal (standard hydrous dextrose) from 2.5% to 4.25%	9.4 (5-14)	Leakage, obstruction	Died
**Noh et al. (2020)**	Sepsis	26+6/7	760	PD catheter/McBurney point	43/Hemosol or Physioneal (standard hydrous dextrose) from 2.5% to 4.25%	9.4 (5-14)	Leakage	Died
**Noh et al. (2020)**	Pulmonary hemorrhage	26+6/7	550	ARROW catheter/McBurney point	3/Hemosol or Physioneal (standard hydrous dextrose) from 2.5% to 4.25%	9.4 (5-14)	Leakage	Died

**Noh et al. (2020)**	Bilateral renal vein thrombosis	23+6/7	470	PD catheter/McBurney point	129/Hemosol or Physioneal (standard hydrous dextrose) from 2.5% to 4.25%	9.4 (5-14)	Leakage	Died
**Noh et al. (2020)**	Bilateral renal vein thrombosis	26+3/7	868	PD catheter/McBurney point	134/Hemosol or Physioneal (standard hydrous dextrose) from 2.5% to 4.25%	9.4 (5-14)	Leakage, intraperitoneal hemorrhage	Died
**Noh et al. (2020)**	Cardiogenic shock, TTTS	27+6/7	590	PD catheter/McBurney point	3/Hemosol or Physioneal (standard hydrous dextrose) from 2.5% to 4.25%	9.4 (5-14)	None	Died
**Noh et al. (2020)**	Sepsis	27+3/7	470	PD catheter/McBurney point	13/Hemosol or Physioneal (standard hydrous dextrose) from 2.5% to 4.25%	9.4 (5-14)	Obstruction	Recovered
**Burgmaier et al. (2020)**	Sepsis	22	430	Ascites drainage catheter	120/ND	19	Hyperglycemia	Died
**Burgmaier et al. (2020)**	Invasive fungal infection	23	614	Drainage catheter-->Acute PD catheter	7-14/ND	44	Leakage, dislocation, peritonitis, obstruction (drainage catheter), hyperglycemia	Survived (CKD4)
**Chen et al. (2021)**	Sepsis	23+5/7	650	ARROW catheter/McBurney point	10/2.5% dialysate (Dianel)	3	Leakage	Recovered

Supplementary Material – Table S1: Case reports of children with a birth weight ≤1000 g and a gestational age ≤28 weeks undergone peritoneal dialysis currently available in literature.
